# International Commission on Trichinellosis: Recommendations on the use of serological tests for the detection of *Trichinella* infection in animals and humans

**DOI:** 10.1016/j.fawpar.2018.e00032

**Published:** 2019-02-05

**Authors:** Fabrizio Bruschi, Maria Angeles Gómez-Morales, Dolores E. Hill

**Affiliations:** a Department of Translational Research, N.T.M.S. Università di Pisa, Via Roma 55, 56126 Pisa, Italy; b Department of Infectious Diseases, Istituto Superiore di Sanità, viale Regina Elena 299, 00161 Rome, Italy; c USDA, ARS, ANRI, APDL, BARC-East, Beltsville, MD 20705, USA

**Keywords:** ELISA, Western blotting, Serology, Anti-*Trichinella* IgG, *Trichinella* infection

## Abstract

Serological methods are widely used for detection of infections in animals and humans. The recommendations provided here take into account the best current methods for the serological detection of *Trichinella* infection. They are based on current scientific information including unpublished data from laboratories with relevant expertise in this field. These recommendations represent the official position of the International Commission on Trichinellosis (ICT) regarding acceptable methods for the use and interpretation of serology testing for *Trichinella* infection in animals and humans.

The ICT does not recommend use of serological methods for testing individual carcasses of animals at slaughter for assuring food safety. For detection of human infections, for epidemiological studies in animals and humans, and for monitoring *Trichinella* infection in swine, the ICT recommends ELISA using excretory/secretory (ES) antigens. These antigens are obtained from the in-vitro maintenance of *Trichinella spiralis* muscle larvae and are recognized by sera from hosts infected by all *Trichinella* species and genotypes identified thus far. In most situations, positive results obtained by ELISA should be confirmed by western blot. Serological assays should be properly standardized and validated for their intended purpose. The components of the test that are critical for maintaining suitable performance should be identified and appropriately checked. Users of commercial tests should verify that the test has been adequately evaluated by an independent body. Serology is useful for detecting *Trichinella* in animals and humans but its limitations need to be taken into account when interpreting the results.

## Highlights

• *Trichinella* serology is not recommended for testing individual animals to assure food safety.
• Serological assays should be standardized and validated for their intended purpose.
• ELISA using excretory/secretory antigens is the test recommended by the ICT.

## 1 Introduction

*Trichinella* spp. are the causative agents of human trichinellosis, a disease that not only is a public health hazard by affecting humans but also represents an economic problem in swine production and food safety. To date, twelve taxa are known and these include encapsulating species *Trichinella spiralis*, *Trichinella nativa*, *Trichinella britovi*, *Trichinella murrelli*, *Trichinella nelsoni*, *Trichinella patagoniensis* and genotypes *Trichinella* T6, T8 and T9 exclusive to mammals, and non-encapsulating species *Trichinella pseudospiralis*, *Trichinella papuae* and *Trichinella zimbabwensis* infecting mammals and birds, or mammals and reptiles (Korhonen et al., 2016).

Serological methods are widely used for detection of infections in animals and humans The recommendations provided here take into account the best current methods for serological detection of *Trichinella* infection in animals and humans and provide guidance on the appropriate use of these serological tools. The International Commission on Trichinellosis (ICT) does not recommend use of serological methods for testing individual carcasses of animals at slaughter for the purpose of assuring food safety (Gamble et al., 2000). This recommendation is consistent with the legislation of many governmental bodies, under which meat inspection programs for *Trichinella* in pork, horse and game meats are performed using a direct method such as artificial digestion (EC, 2015; OIE, 2017; ISO, 2015).

## 2 Assays

Many types of serological assays have been, and continue to be, used for the detection of *Trichinella* infections in animals and man. Serological assays include, but are not limited to:

1) enzyme-linked immunosorbent assay (ELISA) employing excretory-secretory (ES) antigens of the muscle larvae (ML) (Frey et al., 2009a; Gamble et al., 1983, Gamble et al., 2004; Gómez-Morales et al., 2008, Gómez-Morales et al., 2009);
2) immuno-electrotransfer blot assay (IETB), also named western blot (WB), using crude worm extract (CWE) or ES antigens (Frey et al., 2009b; Gómez-Morales et al., 2012, Gómez-Morales et al., 2014; Nöckler et al., 2009; Yera et al., 2003);
3) indirect immunofluorescence assay (IFA), using formalin-fixed whole ML preparations, cryostat sections of infected rodent muscle or frozen sections of free ML (Dupouy-Camet and Bruschi, 2007; Sofronic-Milosavljevic et al., 2005);
4) enzyme immunohistochemical (EIH) technique, employing cryostat sections of infected rodent muscle or frozen sections of free ML (Gamble et al., 2004);
5) lateral flow methods, using immunochromatographic strips and ES antigens (Fu et al., 2013; Zhang et al., 2009).

For detection of infection in swine and humans, ELISA is the most commonly used screening test; positive results should be confirmed by WB (Dupouy-Camet and Bruschi, 2007).

The main advantages of ELISA are high throughput potential, low cost, reliability, standardization, and an acceptable balance between sensitivity and specificity. It is the only serological method in animals recommended by the World Organization for Animal Health (OIE, 2017). For these reasons, the ELISA will be the primary focus of these recommendations. Other types of serological tests can have practical applications; therefore, the principles for use of the ELISA (requirements for performance, suitability for the particular host species, etc.) should be considered in selecting any serological test for detection of *Trichinella* infection.

### 2.1 Antigens

For serological testing by ELISA, the ICT recommends the use of ES antigens obtained from in-vitro maintenance of *Trichinella* ML (Gamble et al., 1983, Gamble et al., 2000, Gamble et al., 2004). This antigen preparation contains a group of immunodominant, structurally-related glycoproteins that are recognized by animals and humans infected with *Trichinella spiralis*, or any of the other species of *Trichinella* currently known (Appleton et al., 1991; Ortega-Pierres et al., 1996). When compared with somatic worm extracts, these antigens have limited cross-reactivity with sera from animals infected with other parasites (Davidson et al., 2009; Gamble et al., 2004; Gómez-Morales et al., 2009, Gómez-Morales et al., 2012; Møller et al., 2005; Nöckler and Kapel, 2007; Nöckler et al., 2004; Szell et al., 2012). Numerous methods have been published for the preparation of ES antigens; however, for consistency among preparations and reproducibility of ELISA data, the ICT recommends only that method published in the OIE Manual of Diagnostic Tests and Vaccines for Terrestrial Animals (OIE, 2017). *Trichinella* ES antigens are routinely prepared from *T. spiralis* ML because this species is readily maintained in laboratory animals, and these antigens are recognized by sera from hosts infected by all *Trichinella* species and genotypes identified thus far (Appleton et al., 1991).

### 2.2 Reagents

To maximize test sensitivity and specificity, it is recommended that a species-specific anti-IgG conjugate rather than a Protein A (or similar) conjugate be used in the ELISA or WB (Gamble et al., 2004).

### 2.3 Sample collection

Serum is the preferred sample material for indirect detection of *Trichinella* infection using serology. After collection, blood samples should be clotted, sera collected, and, if not used for testing immediately, frozen at −20 °C. Samples frozen at −20 °C may be used for several months; however, it is recommended that repeated freezing and thawing of samples should be avoided in order to prevent antibody degradation and an increase in non-specific reactivity. If serum samples are to be used frequently, they should be stored in aliquots. For periods of storage >3 months, serum samples should be frozen at −80 °C or lyophilized. If freezing is not possible, 1% merthiolate (used at 1:10,000 dilution) or another suitable preservative should be added (OIE, 2013; Harlow and Lane, 1988).

An alternative to conventional use of blood or serum samples is blood spots on filter paper. This method is useful when there are no facilities to store frozen samples (Owen et al., 2005; Vu Thi et al., 2010). Blood spots may be stored at room temperature in closed plastic bags to prevent rehydration.

For tests performed on animal carcasses, where blood or serum is not available, tissue fluids can be used as alternative sources of antibody (Gamble et al., 2004). Usually, samples of tissue fluids are used at a lower dilution (higher concentration) in serological assays as antibody concentration in tissue fluids may be 10-fold lower than that found in serum (Gómez-Morales et al., 2014; Kapel et al., 1998; Møller et al., 2005). When meat samples are used for the extraction of tissue fluids it is recommended to wash the tissue, blot with paper towel to remove excess water, cut it into small pieces, freeze and thaw it and use the extracts thus obtained.

## 3 Validation and quality control

An acceptable serological assay should be properly standardized and validated for its intended purpose. The components of the test that are critical for maintaining suitable performance (critical control points) should be identified and appropriately monitored. Furthermore, the test should be conducted within a laboratory quality system. In particular, each batch or lot of antigens should be evaluated by checkerboard titration using standardized positive and negative control sera.

Requirements for the development and validation of a serological test in animal populations are specified by the OIE (OIE, 2013). Users of commercial tests should verify that the test has been adequately evaluated using international reference standards and has received the approval of relevant regulatory authorities. It is important that users of any test conduct an ‘in-house’ verification of test performance, using panels of defined positive and negative sera representative of the target population whenever feasible.

## 4 Use of serology in animals

Animal hosts can harbor infective ML as early as 18 days post infection (Despommier, 1998), in some cases before detectable antibodies are present; further, infection with low numbers of larvae can result in an extended period of seronegativity before anti-*Trichinella* antibody is detectable in serum (Nöckler et al., 2005). It has been reported that the correlation between seropositivity and the presence of *Trichinella* ML decreases at low infection rates. For these reasons, serological methods should not be used for the detection of *Trichinella* infection in individual food animal carcasses for the purpose of protecting human health (Gajadhar et al., 2009; Gamble et al., 2004).

### 4.1 ELISA for detection of Trichinella infection in domestic swine populations

#### 4.1.1 Suitability of test

ELISA, due to its ease of use, low cost, rapidity in obtaining results, and potential for standardization and automation for large numbers of samples, is the test of choice for surveillance in domestic pigs. ELISA, using ES antigens, has been shown to have greater sensitivity than digestion of 1 g samples in animals with low (i.e. <3 larvae per gram (lpg)) worm burdens (Gamble et al., 2000; Kapel and Gamble, 2000). However, this increased sensitivity, as compared with direct testing methods, is offset by the reduced ability to detect antibodies in recently infected animals, even when infective larvae are found in the muscle. Thus, ELISA is not advised for individual carcass control. However, ELISA is an excellent tool for epidemiological studies and for monitoring *Trichinella* exposure.

#### 4.1.2 Validation of ELISA

Serological detection of *Trichinella* infection in pigs is impacted by both technical (laboratory proficiency, quality of the antigen used in the assay) and biological factors (initial infecting dose, days post-infection). Prior to using ELISA for detection of antibodies to *Trichinella*, the test should be fully validated with an appropriate number of positive and negative samples from the test population (OIE, 2013). Validation should take into account that false negatives can occur during a period of prolonged seroconversion due to a low infectious dose or low larval density in muscle tissue or from collection of serum before a detectable antibody response has developed. False positives can occur from non-specific serological reactivity to components in a complex antigen preparation, or to cross-reacting antibodies generated from a different helminth infection. This is particularly evident in free-ranging and backyard pigs which are also at higher risk for *Trichinella* sp. infection. Therefore, positive results should be confirmed by WB (Dupouy-Camet and Bruschi, 2007).

#### 4.1.3 Antigen preparation

The quality of ES antigens used in the ELISA is of primary importance, and depends upon adherence to proper methods for the cultivation of *Trichinella* ML and proper purification and storage of the antigen. The method for the preparation of ES antigens has been published in the OIE Manual (OIE, 2017).

#### 4.1.4 Methodologies and controls

A general method for conducting an ELISA test in pigs is described in the OIE Manual (OIE, 2017). Standard antigens, reference sera and scientific consultation can be obtained from subject matter authorities, such as ICT members' laboratories (www.trichinellosis.org) and the OIE Reference Laboratories for Trichinellosis (www.oie.int/eng/oie/organisation/en_listeLR.htm). Reference swine sera positive for anti-*Trichinella* antibodies are not available on the international market; however, swine sera from experimentally infected animals have been collected and their validity and stability tested (Gómez-Morales et al., 2015). These reference sera are available upon request at the European Union Reference Laboratory for Parasites (http://www.iss.it/crlp/).

#### 4.1.5 Interpretation of results

The level of infection of pigs with *Trichinella* larvae (worm burden) is directly correlated with the time required for anti-*Trichinella* antibodies to appear in the blood. For low-grade infections (< 1 lpg), antibodies may not be detected by ELISA for 4–7 weeks or longer following exposure (Gamble, 1996, Gamble, 1998; Gamble et al., 1983), while antibodies might be detected after 2.5 to 3 weeks in pigs with higher numbers of ML. There is no correlation between the ultimate worm burden (larvae per gram of tissue) and the resulting optical density (OD) in the ELISA in serologically positive pigs once seroconversion has taken place. Therefore, artificial digestion of tissue is an important adjunct to ELISA to determine the public health risk associated with infected animals. *Trichinella* antibodies may persist in pigs for extended periods. It can be assumed that in slaughter pigs, which have a live weight of 90 to 100 kg at an age of 25 to 30 weeks, it is unlikely that a false-negative finding would result from declining antibody titer.

### 4.2 Indirect detection of Trichinella infection in other animals, including wildlife

Several ELISAs to monitor wildlife populations such as wild boar have been described; however, the c-ELISA that enables the detection of specific antibodies irrespective of their isotype or host origin, has the most potential value as a multispecies surveillance tool (Gamble and Graham, 1984; Gnjatovic et al., 2017). The variability of collection methods for wildlife and game meat serum samples often creates problems in conducting serological tests. Samples are frequently contaminated by bacteria or fungi, or they may be hemolysed; these problems can cause false positive results (OIE, 2013; Harlow and Lane, 1988). Besides the compromised quality of the samples, the validation of serological assays is also hampered by a lack of reliable reference sera. Any serological test used to detect *Trichinella* infection in animal species other than pigs should likewise be fully validated. As for domestic pigs, a confirmatory test, such as WB, for positive sera should be performed. Examples of ELISA performance in animal species other than domestic swine are presented in Table 1.

**Table 1:** Performance of ELISA with ES antigens in animal species other than domestic swine.

Animal species	Notes	References
Horse (Equus caballus)	Antibody responses persisted in a dose-dependent manner from 14 to 20 weeks post-infection (p.i) and then declined to undetectable levels, whereas, viable ML persisted in horse muscle for longer period of time	Hill et al., 2007; Nöckler et al., 2000; Pozio et al., 2002
Dog (Canis lupus familiaris)	ELISA followed by a confirmatory Western blot using ES antigens have been developed and validated; no commercial kit is available	Gómez-Morales et al., 2016
Wild boar (Sus scrofa)	Results similar to those of domestic pigs but with a higher number of false positives	Cuttell et al., 2014; Gómez-Morales et al., 2014; Kärssin et al., 2016
Bear (Ursus spp.)	Lack of reference sera and validation studies	Asbakk et al., 2010; Mortenson et al., 2014; Rah et al., 2005
Fox (Vulpes spp.)	Lack of reference sera and validation studies	Davidson et al., 2009; Nöckler and Voigt, 1998
Crocodile (Crocodylus niloticus)	Antibodies were not detectable after six weeks p.i. although live larvae were present in the muscles up to six months p.i.	Ludovisi et al., 2013
Seal (Halichoerus grypus)	Specific antibody levels increased during the 10 week experimental period. Very reduced number of animals.	Kapel et al., 2003

#### 4.2.1 Interpretation of results

It is imperative to determine the positive/negative cut-off value and associated sensitivity and specificity on the basis of a panel of serum samples (at least 100–200 sera representative of the animal population for which the test will be used). Alternative methods such as a binary mixed model analysis, which was shown effective for other animal parasitic diseases, are not feasible at a low expected prevalence of the infection. The animal genotype, feeding habits, exposure to other pathogens, and environmental characteristics can influence the background of a serological test (OIE, 2013). All these factors are particularly relevant in wildlife and other animal species that are not raised under controlled conditions.

## 5 Use of serological methods in humans

Since there are no pathognomonic signs or symptoms for trichinellosis, clinical diagnosis in individuals is often difficult. Consequently, diagnosis is based on three main criteria: anamnesis based on epidemiological data, clinical evaluation, and laboratory tests including serology and/or the detection of *Trichinella* larvae in a muscle biopsy (Dupouy-Camet and Bruschi, 2007). Because the collection of a muscle biopsy is invasive, painful, and does not always give the expected result even when the suspicion of trichinellosis is correct, serological findings, normally entailing the detection of specific IgG in serum, have practical diagnostic value.

There are 3 objectives in the immunodiagnosis of human trichinellosis: (a) recognizing the acute infection to allow early anthelminthic treatment; (b) making a retrospective diagnosis; and (c) contributing information to the epidemiology of the infection (Ljungström, 1983).

### 5.1 Suitability of test

Many serological tests are available for human diagnosis (Dupouy-Camet and Bruschi, 2007). ICT recommends the use of an ELISA for screening and WB to confirm ELISA-positive sera. All tests should use ES antigens. Serological diagnosis can be complicated by cross-reactivity, due to the presence of shared antigens of *Trichinella* spp. in other parasites and pathogens (Gómez-Morales et al., 2008; Intapan et al., 2006).

In most trichinellosis cases, increased parasite-specific IgG, IgA and IgM serum levels accompany the infection; however, increases in parasite-specific IgE antibody and total IgE are not consistent, and consequently the diagnostic value of IgE antibodies without considering other laboratory findings is limited (Bruschi and Dupouy-Camet, 2014).

Generally, seroconversion occurs between the third and fifth weeks of infection and antibody levels do not correlate with the severity or other aspects of the clinical course. IgG specific antibodies are detectable from 12 to 60 days post infection and may persist for >30 years after infection (Bruschi and Gómez-Morales, 2014; Bruschi et al., 2005). The identification of IgG subclasses, although interesting for research purposes, does not contribute to the diagnosis (Pinelli et al., 2004, Pinelli et al., 2007). For interpreting human serology in the course of *Trichinella* infection, the Food and Agriculture Organization (FAO)/World Health Organization (WHO)/World Organization for Animal Health (OIE) guidelines for the surveillance, management, prevention and control of trichinellosis should be consulted (Dupouy-Camet and Bruschi, 2007).

An example of a detailed protocol for performing an ELISA with human sera is shown in Appendix A.

## 6 Conclusion

These recommendations are based on current scientific information including unpublished data from laboratories with relevant expertise in this field. They represent the official position of the ICT regarding acceptable methods for the use and interpretation of serology testing for *Trichinella* infection in animals and humans. These recommendations are subject to change as new scientific information becomes available.

The following is the supplementary data related to this article.

Appendix A Procedure for the detection of anti-*Trichinella* antibodies in human serum by indirect ELISA Appendix A

## Conflict of interest

The authors declare no conflict of interest.
