# Supplementary material for: International Commission on Trichinellosis: Recommendations on the use of serological tests for the detection of Trichinella infection in animals and humans
Source: Food Waterborne Parasitol. 2019 Feb 5;14:e00032. doi: 10.1016/j.fawpar.2018.e00032 (PMC7034015; doi:10.1016/j.fawpar.2018.e00032)
Supplement: Appendix A — Procedure for the detection of anti-Trichinella antibodies in human serum by indirect ELISA [file mmc1.doc]

**Appendix A**

**Procedure for the detection of anti-*Trichinella* antibodies in human serum by indirect ELISA**

1. **Aim and field of application**

To determine the presence of anti-*Trichinella* sp. antibodies by an enzyme linked immunosorbent assay in human sera.

The method can be used for the serological diagnosis of human trichinellosis.

1. **Principle of the method**

A 96-well microtiter polystyrene plate is coated with *Trichinella* *spiralis* excretory/secretory (E/S) antigens.

Control and test sera, properly diluted, are distributed in the wells, allowing any anti-*Trichinella* sp. antibodies that are present to bind to the adsorbed antigen.

The antibodies that do not bind to the antigen are eliminated by washing; peroxidase conjugated anti-human IgG goat antibody is then added to each well. This second incubation allows the conjugate to bind to the human antibodies that were bound to the antigens onto the well surface.

The excess conjugate is eliminated by washing, and the activity of the enzyme bound to the human antibodies is measured by adding a chromogen substrate. After incubation, the intensity of the developed color is determined by a spectrophotometer.

The result is interpreted comparing the color intensity of the wells containing the test sera with those containing the controls.

1. **References**

Centers for Disease Control, Office of Health and Safety, www.cdc.gov/od/ohs/biosfty/bmbl4/b4af.htm

Gamble HR, Pozio E, Bruschi F, Nockler K, Kapel CM, Gajadhar AA., 2004. International Commission on Trichinellosis: recommendations on the use of serological tests for the detection of *Trichinella* infection in animals and man. Parasite.;11, 3-13.

Gómez-Morales MA, Ludovisi A, Amati M, Cherchi S, Pezzotti P, Pozio E., 2008. Validation of an enzyme-linked immunosorbent assay for diagnosis of human trichinellosis. Clin Vaccine Immunol. 15,1723-9.

1. **Definitions**

ELISA Enzyme Linked Immunosorbent Assay

Ag Antigen

Ab Antibodies

Ag E/S Excretory/Secretory antigens

BSA Bovine Serum Albumin

PBS Phosphate Buffered Saline

H Hours

Min Minutes

RT Room temperature

1. **Devices/instruments**

The following instruments are needed to prepare the reagents to perform the ELISA procedure.

- 1. Adjustable pipettes (volumes: 1 - 1000 μL)
  2. Balance (0.01-100gr)
  3. Automatic plate washer (strongly recommended)
  4. ELISA plate microtiter reader
  5. Freezer -20/-30°C
  6. Ice maker
  7. Incubator 37°C
  8. Magnetic stirrer
  9. Adjustable volume dispenser (e.g., Multipette Eppendorf®)
  10. pH meter
  11. Pipette aid
  12. Refrigerator +4°C ± 2°C
  13. Vortex

**6. Reagents and chemicals**

The step-by-step procedure for preparing the reagents is described below.

- 1. Analytical grade water
  2. Phosphate buffered saline (PBS), pH 7.3 ± 0.2

KH_2_PO_4_ 0.34 g

Na_2_HPO_4_ 1.21 g

NaCl 8.0 g

Analytical grade water up to 1000 mL

Dissolve compounds in 750 mL of analytical grade water under magnetic stirring. Check the pH (7.3 ± 0.2) and then bring the solution to the final volume; refrigerate.

6.3 Carbonate buffered saline, pH 9.6 ± 0.2

Na_2_CO_3_ 1.12g

NaHCO_3_ 2.92g

Analytical grade water up to 1000 mL

Dissolve the compounds in 750 mL of analytical grade water under magnetic stirring. Check the pH (9.6 ± 0.2) and then bring the solution to the final volume; store at room temperature. If needed, clear the solution by filtration.

6.4 Washing solution

### Tween 20 1 mL

Analytical grade water up to 2000 mL

The solution should be prepared immediately before use, as follows: add 1 mL of Tween 20 to a 2 L flask; bring the solution to the final volume by adding analytical grade water and mix by magnetic stirring until the solution is clear. If refrigerated, the solution should be used within 24 h.

6.5 Blocking solution

### BSA 0.25 g

Tween 20 0.05 mL

PBS up to 50.00 mL

The solution should be prepared immediately before use, as follows: place 0.25 g BSA (bovine serum albumin) directly in a 50 mL tube; add 40 ml of PBS buffer and mix by vortexing until the BSA is completely dissolved. Add 0.05 mL Tween 20; mix by vortexing and bring to volume. If refrigerated, the solution must be used within 24 h.

6.6 Sera and conjugate diluent

BSA 1.00 g

Tween 20 0.05 mL

### PBS up to 100 mL

The solution should be prepared immediately before use, as follows: place 0.50 g BSA directly in a 50 mL tube; add 40 ml of PBS buffer and mix by vortexing until BSA is completely dissolved. Add 0.025 mL Tween 20; stir by vortexing and bring it to volume. If refrigerated, the solution must be used within 24 h.

6.7 Stop solution

HCl 1N in analytical grade water. Prepare the solution under a chemical hood; store at room temperature.

6.8 TMB (3, 3’, 5, 5’ tetramethylbenzidine) peroxidase substrate

This substrate is recommended; if not available, any other peroxidase substrate can be used.

6.9 96-well flat bottomed microtiter plate

6.10 Excretory/secretory antigens (ES Ag) (see OIE Manual, http://www.oie.int/fileadmin/Home/eng/Health_standards/tahm/2.01.20_TRICHINELLOSIS.pdf )

The antigens at the appropriate concentration (for example 5µg/mL) should be brought to a final volume of 12 mL with carbonate buffer saline pH 9.6. The dilution should be performed on ice immediately before use.

6.11 Peroxidase labelled anti–human IgG goat antibodies

The conjugate should be used at the optimal dilution calculated by checking board titration versus a standardised positive control serum. The dilution should be prepared on ice immediately before use.

6.12 Anti-*Trichinella* sp. seropositive control sera

100 μL of diluted sera from *Trichinella* sp. infected persons (positive controls). Each positive control serum should be properly diluted (e.g., 1/200), using the appropriate diluter. The dilution should be performed on ice immediately before use.

6.13 Anti-*Trichinella* sp. negative control sera

100 μL of diluted sera from *Trichinella* sp. free persons (negative controls). Each negative control serum should be properly diluted (e.g., 1/200), using the appropriate diluter. The dilution should be performed on ice immediately before use.

6.14 Sera to be tested

Each serum should be tested at the same dilution that control sera, using the appropriate diluent. The dilution should be performed on ice immediately before use.

# Procedure

- 1. Preparing test and control samples

7.1.1 Thaw the test sera and the positive and negative control sera by storing them at +1-8°C for at least 5 h.

7.1.2 Once thawed, keep them in an ice bath and stir them by vortexing before use.

7.1.3 Dilute 1:200 the test and control sera as follows: in a 1-2 mL conical bottom tube, add 5 µL of serum and 995 µL diluting solution. Diluted sera can be stored refrigerated for up to 24 h.

- 1. Analytical procedure.

7.2.1 Fill the microtiter plate with 100 μL per well of ES Ag in carbonate buffered saline; incubate for 1h at 37°C.

7.2.3 Wash 3 times in the automatic plate washer with the washing solution.

7.2.4 Add 200 μL blocking solution per well; incubate for 1 h at 37° C.

7.2.5 Wash 3 times in the automatic plate washer with the washing solution.

7.2.6 Add 100 μL of each diluted sample per well and incubate for 30 min at 37°C.

7.2.7 Each serum dilution should be performed in duplicate.

7.2.8 Sera should be diluted (e.g., 1/200).

7.2.9 Wash 3 times in the automatic plate washer with the washing solution.

7.2.10 Add 100 μL of the diluted anti–human IgG peroxidase labelled antibodies per well and incubate for 1 h at 37°C.

7.2.11 Wash 3 times in the automatic plate washer with the washing solution.

7.2.12 Add 100 μL TMB substrate per well; incubate for 10 min at room temperature.

7.2.13 Stop the reaction by adding 50 μL of the stop solution per well and read the reaction in the ELISA plate microtiter reader at 450 nm.

1. **Interpretation of the results**

8.1 The test results can be considered as valid if all of the following criteria are fulfilled:

8.1.1 The OD value of the negative control sera should be lower than the *cut off* value determined during the validation process of the method

- - 1. The OD value of the positive control sera has to be higher than the *cut off* value determined during the validation process of the method;
    2. The difference in OD between the 2 measures made on the same positive control sample in strict conditions of repeatability has to be < 0.15 unit absorbance, and on the same negative control sample it has to be < 0.05 unit absorbance.

If even only one of the above-reported criteria is not met, the test has to be considered as non-valid and the sera should be tested again.

- 1. Calculate the mean of the 2 duplicates for each positive sera (PS) and for each test sera (TS).
  2. Subtract from each mean value the mean OD value of the blanks (OD_b_).
  3. Select the higher OD value among the positive control sera (PS_max_), and for each sample calculate the extinction value (I_e_) according to the following formula:

OD mean duplicates TS – OD_b_

Ie (%) = ___________________________________ X 100%

OD mean duplicates highest PS – OD_b_

where: Ie > 11.8%, *Trichinella* positive serum

Ie < 11.8%, *Trichinella* negative serum

**9. Safety measures**

This method should be carried out only by authorized personnel. The operator should wear personal protection equipment (PPE) during the test performance. For the general safety measures, refer to the guidelines of CDC.
